# A proline metabolism selection system and its application to the engineering of lipid biosynthesis in Chinese hamster ovary cells

**DOI:** 10.1016/j.mec.2021.e00179

**Published:** 2021-07-27

**Authors:** James D. Budge, Joanne Roobol, Gurdeep Singh, Théo Mozzanino, Tanya J. Knight, Jane Povey, Andrew Dean, Sarah J. Turner, Colin M. Jaques, Robert J. Young, Andrew J. Racher, C. Mark Smales

**Affiliations:** a Industrial Biotechnology Centre, School of Biosciences, University of Kent, Canterbury, Kent CT2 7NJ, UK; b Lonza Biologics, 228 Bath Road, Slough, SL1 4DX, UK; c Cell Engineering Group, Lonza Biologics, Chesterford Research Park, Building 200, Little Chesterford, CB10 1XL, UK

**Keywords:** Chinese hamster ovary (CHO) cells, Metabolic selection system, Cell engineering, Proline metabolism, Biotherapeutic protein production

## Abstract

Chinese hamster ovary (CHO) cells are the leading mammalian cell host employed to produce complex secreted recombinant biotherapeutics such as monoclonal antibodies (mAbs). Metabolic selection marker technologies (e.g. glutamine synthetase (GS) or dihydrofolate reductase (DHFR)) are routinely employed to generate such recombinant mammalian cell lines. Here we describe the development of a selection marker system based on the metabolic requirement of CHO cells to produce proline, and that uses pyrroline-5-carboxylase synthetase (P5CS) to complement this auxotrophy. Firstly, we showed the system can be used to generate cells that have growth kinetics in proline-free medium similar to those of the parent CHO cell line, CHOK1SV GS-KO™ grown in proline-containing medium. As we have previously described how engineering lipid metabolism can be harnessed to enhance recombinant protein productivity in CHO cells, we then used the P5CS selection system to re-engineer lipid metabolism by over-expression of either sterol regulatory element binding protein 1 (SREBF1) or stearoyl CoA desaturase 1 (SCD1). The cells with re-engineered proline and lipid metabolism showed consistent growth and P5CS, SCD1 and SREBF1 expression across 100 cell generations. Finally, we show that the P5CS and GS selection systems can be used together. A GS vector containing the light and heavy chains for a mAb was super-transfected into a CHOK1SV GS-KO™ host over-expressing SCD1 from a P5CS vector. The resulting stable transfectant pools achieved a higher concentration at harvest for a model difficult to express mAb than the CHOK1SV GS-KO™ host. This demonstrates that the P5CS and GS selection systems can be used concomitantly to enable CHO cell line genetic engineering and recombinant protein expression.

## Highlights

We have engineered a proline P5CS metabolism selection system in CHO cells
P5CS proline selection was used to engineer lipid metabolism in CHO cells
P5CS selection was stable for at least 100 generations
P5CS and GS selection systems were used together to engineer lipid and mAb expression
Lipid metabolism P5CS engineered CHO cells give enhanced recombinant protein expression

## 1 Introduction

Recombinant protein drugs are manufactured using a range of expression systems. Chinese hamster ovary (CHO) cells are the expression system most widely used to produce complex human-like recombinant proteins such as monoclonal antibodies (mAbs) (Walsh, 2018). As such, a kaleidoscope of processes and technologies required for the generation and purification of recombinant material from CHO cells has been explored to improve productivity, ensure consistent product quality and reduce process timelines (Vito et al., 2020; Povey et al., 2014; Pekle et al., 2019; Shukla et al., 2018; Budge et al., 2021). Yet there remains a need and desire to further improve such expression systems, particularly for so termed difficult to express (DTE) proteins (Roobol et al., 2020).

Selection markers and the type of marker are a key technology for isolating CHO cells expressing transgenes of interest and ensuring their continued expression with increasing cell age. Initially, the markers were eukaryotic proteins e.g. neomycin phosphotransferase that allowed the transfected cell to grow in the presence of the cognate antibiotic. There are problems with antibiotic-based selection systems: antibiotics can alter host cell gene expression that may impact productivity yields and/or product quality (Ryu et al., 2017), mask low-level contamination with adventitious agents, the antibiotic itself is a contaminant in the manufacturing process (Mignon et al., 2015; Davies, 1996) that must be removed from the drug substance, and their use is not desirable in a GMP facility due to the health risks arising from potential cross-contamination.

An alternative approach to antibiotic selection is to use metabolic selection markers. Dihydrofolate reductase (DHFR) (Kaufman and Sharp, 1982a; Jossé et al., 2018) and glutamine synthetase (GS) (Cockett et al., 1990; Birch et al., 2008) are well established examples of metabolic selection. DHFR reduces dihydrofolate to produce tetrahydrofolate, a vitamin required as a precursor for *de novo* synthesis of essential molecules such as hypoxanthine and thymidine (Kaufman and Sharp, 1982b). In the absence of exogenous hypoxanthine or thymidine DHFR-deficient CHO host cells are unable to survive. However, introduction and expression of the *DHFR* gene can restore the ability of a cell to produce sufficient tetrahydrofolate and, in turn, hypoxanthine and thymidine therefore enabling cells to survive with no external source of these essential molecules. Similarly, GS is the enzyme responsible for the conversion of glutamate to glutamine. CHO glutamine-auxotrophs, usually created by the inhibition of endogenous GS by methionine sulfoximine (MSX) (Feary et al., 2017) or knockout of endogenous GS (Fan et al., 2012), are unable to survive in a glutamine-free environment. However, by introducing an exogenous glutamine synthetase (*GLUL*) gene cells can produce sufficient glutamine to survive without an external glutamine source (Bebbington et al., 1992). The auxotrophic nature of CHO cells based around the inability to produce hypoxanthine and thymidine or glutamine has enabled exploitation of *DHFR* and *GLUL* genes respectively to select for stably expressing cells. As outlined above, in order to improve the selection stringency of both of these systems, inhibitors are often introduced to the culture medium which inactivate the enzymatic function of the selection gene. Methotrexate (MTX) and MSX are used to inhibit the activity of DHFR or GS respectively, and only cells expressing appropriate amounts of these enzymes to overcome the inhibitory concentrations are able to grow.

The DHFR and GS systems are well established in industry for the generation of recombinant biotherapeutic protein producing CHO cell lines. However, with the development of more complex non-natural molecules made from multiple gene products together with interest in synthetic biology approaches for the engineering of production hosts to enhance their ability to produce higher amounts and quality of proteins, there is a need to develop additional, non-antibiotic based metabolic selection systems. This would allow the engineering of host cells using one selection marker whilst leaving the GS or DHFR selection marker available to subsequently generate and select for recombinant biotherapeutic protein producing cells. A further application of new metabolic markers would be for the development of improved bioprocesses that do not require particular metabolites to be present in medium or feed, simplifying the bioprocess. Indeed, there have been a number of reports of such alternative selection systems. For example, Zhang et al. reported the development of a double auxotrophic selection system based on knockout of two enzymes involved in pyrimidine and purine biosynthesis and utilised these for selection and isolation of cell lines expressing a model Fc-fusion and mAb molecule (Zhang et al., 2020). In a different approach, Capella Roca et al. have reported the development of an arginase based selection system for CHO cell hosts lacking arginase activity (Roca et al., 2019). Very recently, Sun et al. reported a proline based metabolic selection system used to isolate GFP and mAb expressing recombinant CHO cell lines, and although the mAb titres were not high by industrial standards (approximately 3.4 μg/ml) they were comparable to those achieved from antibiotic selection systems including zeocin (2.25 μg/ml) and G418 (1.65 μg/ml) based systems (Sun et al., 2020). We note that the high mAb titres achieved industrially with other metabolic selection systems (e.g. DHFR, GS) have access to, and utilise, highly optimised host cells, expression vectors, and inhibitors to improve selection stringency alongside refined culture processes. Typically, media for sustaining mammalian cell growth contain proline because such cells (including CHO strains derived from CHO–K1) are unable to synthesise sufficient proline to sustain necessary cellular processes such as *de novo* protein synthesis required to support rapid cell proliferation (Sahu et al., 2016).

Here we describe the development of a pyrroline-5-carboxylase synthetase gene selection marker system, application of P5CS inhibitors to the selection process and its application to the engineering of lipid metabolism in CHO cells. Pyrroline-5-carboxylase synthetase (P5CS) is a key enzyme involved in the synthesis of proline from glutamate (Pérez-Arellano et al., 2010). Expression of transgenic P5CS allows cells with sufficient P5CS to synthesise the required amounts of proline from glutamate to survive in the absence of exogenous proline, thus facilitating selection of desired cells. We then successfully employed the selection system to facilitate genetic over-expression engineering of either sterol regulatory element binding protein 1 (SREBF1) or stearoyl CoA desaturase 1 (SCD1) lipid modifying genes in the CHOK1SV GSKO™ host cell line. We have previously shown how manipulation of these lipid-altering genes can improve cellular processes involved in recombinant therapeutic expression (Budge et al., 2019, 2020). Importantly, we show that the utilisation of the P5CS based system does not affect the subsequent use of the GS based selection system to generate recombinant biotherapeutic expressing CHO cell lines producing industrially relevant amounts of recombinant protein. This shows that the P5CS and GS systems can be used concurrently to enable CHO cell line genetic engineering and recombinant biotherapeutic protein cell line construction processes.

## 2 Methods

### 2.1 Vector construction

The *CgPICR_007864* gene (for P5CS) was obtained by commercial synthesis (GeneArt, ThermoFisher Scientific, USA) such that the amino acid sequence shared 100% identity with the CHO cell derived sequences (accession number XM_007631963.2). The nucleotide sequence was based upon the corresponding mRNA sequence (XM_003508062.2) with a small number of nucleotides changed to facilitate subsequent cloning ensuring that the amino acid sequence was not altered. The gene was cloned into Lonza proprietary expression vectors using *Bgl*II and *Nhe*I restriction enzymes such that expression of the gene is driven by an SV40 promoter. A combination of Gibson Assembly (NEB) and restriction cloning was used, as per the manufacturer's instructions, to generate vectors which comprised the P5CS expression cassette in combination with expression cassettes for either *eGFP*, stearoyl CoA desaturase (*SCD1*) or sterol regulatory element binding factor 1 (*SREBF1*). The source of the *SCD1* and *SREBF1* genes has been previously described (Budge et al., 2019). Different promoters, considered by the authors to give relatively low (PGK promoter), mid (SV40 promoter) or high (mCMV) transcriptional activity, were employed to drive different levels of expression of the *SCD1* and *SREBF1* genes. The mCMV promoter was consistently employed to drive *eGFP* gene expression.

### 2.2 Mammalian cell culture

The Lonza CHOK1SV GSKO™ host cell line (a GS knockout cell line with no endogenous GS expression) was used exclusively throughout this study. Cell concentrations and culture viability data were determined using a ViCell (Beckman Coulter) instrument. The medium used was either commercial Gibco CD-CHO medium (ThermoFisher Scientific) or Medium A (Lonza proprietary medium) as described in the text. Bespoke L-proline-free CD-CHO based medium was prepared by ThermoFisher Scientific. For routine culture, suspension cultures were seeded at 0.2 × 10^6^ viable cells/ml in 20 ml in a 125 ml Erlenmeyer flasks (Corning) and incubated at 37 °C. For 96 deep well plate experiments, these were cultured at 37 °C in a total volume of 300 μl per well. Cells were also grown in an Ambr®15 platform (Sartorius Stedim Biotech) using a proprietary fed-batch process for GS-CHO cell lines (Lonza).

### 2.3 Occurrence of spontaneous revertants

To determine an estimate of the frequency with which spontaneous proline-independent revertants occur, 96 well plates were seeded at either 5000 or 1000 viable cells per well in a volume of 200 μl in different media. Fifteen plates at each cell concentration were seeded in either CD-CHO medium supplemented with 6 mM L-glutamine, CD-CHO with no L-glutamine or Medium A supplemented with L-glutamine and lacking L-proline. The number of colonies (defined as an obvious group of cells clustered in an area) that grew was determined by visual inspection using a light microscope, and the result expressed as a percentage of total cells seeded.

### 2.4 Electroporation to transfect plasmid DNA into cells

Electroporation was used to introduce vector DNA into CHO cells. The DNA solution, 20 μg of DNA in 100 μl of TE buffer, was mixed with 1 × 10^7^ viable cells in 700 μl of culture medium using a 4 mm electroporation cuvette (BioRad). A pulse was applied using the exponential protocol on a GenePulser Xcell electroporator (Bio-Rad) instrument at 300 V and 900 μF.

### 2.5 Cell line construction

Vector DNA constructs (50 μg) for cell line construction were linearised using the *Pvu*I (NEB) restriction enzyme as previously described (Budge et al., 2019). Linearised DNA (20 μg) was then transfected into the CHOK1SV GS-KO™ host cell line as in section 2.4. Transfected cells were then transferred to static T75 flasks and sufficient medium added to give a total volume of 20 ml of the appropriate medium (e.g. L-proline-free medium was used to generate cell pools using a construct bearing the *CgPICR_007864S* gene).

### 2.6 P5CS inhibitors

L-Azetidine-2-carboxylic acid (*Sigma A0760*) and 3,4-Dehydro-L-proline (*Sigma D4893*) are known inhibitors of P5CS and were evaluated in the proline based selection system (Fig. 4). The compounds were dissolved directly in proline-free Medium A (Lonza) to achieve the desired concentration. A range of concentrations (between 1 and 20 mM) were initially screened (data not shown, when inhibitor concentrations were too high cells did not survive beyond the 24 h timepoint) in order to select the concentrations used to evaluate the inhibitors. The initial concentration range was selected based on the working concentration of ornithine (an analogue of both inhibitors) which has previously been reported to be effective in inhibiting P5CS activity at 5 mM (Hu et al., 1999).

**Fig. 1:**
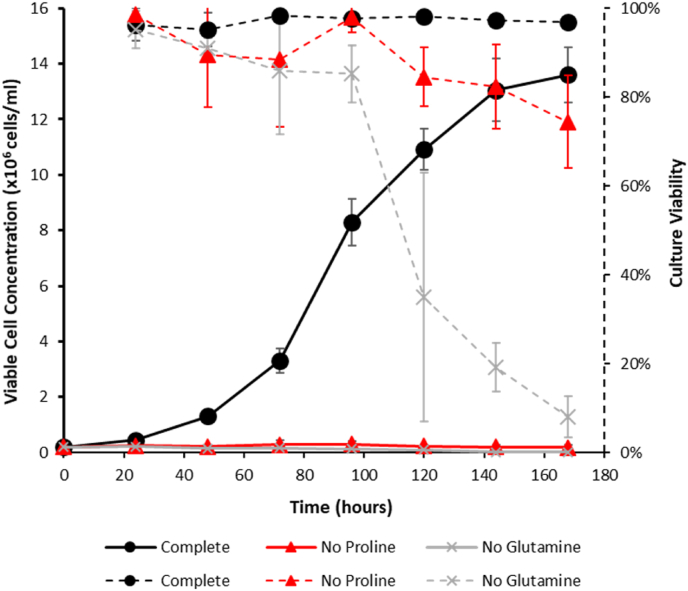
Growth profiles showing viable cell concentration and culture viability of CHOK1SV GSKO™ cells in CD-CHO medium including L-proline and L-glutamine (complete) or L-proline-free or L-glutamine-free medium. Solid lines show the viable cell concentration whilst broken lines show culture viabilities. Batch cultures were seeded at 0.2 × 10 (Budge et al., 2021) viable cells/ml in a total culture volume of 20 ml and cell concentrations measured using a ViCell every 24 h. The value at 0 h was calculated based on the concentration used to set up the initial cultures. N = 3 for each data point and error bars represent ± one standard deviation.

**Fig. 2:**
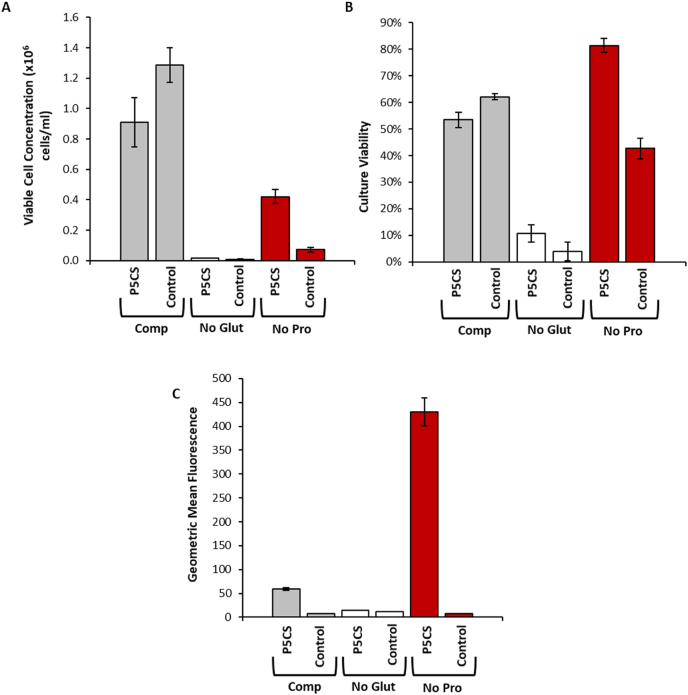
Initial recovery of CHOK1SV GSKO™ cells transiently transfected with vectors bearing either *GS, CgPICR_007864* (for P5CS) or no (Control) selection genes as well as the *eGFP* reporter gene. Cells were transfected on day 0 and data was collected 7 days after transfection having been cultured in the specified medium. Comp (complete) medium contained glutamine and proline, ‘no glut’ had no glutamine present in the medium, ‘no pro’ had no proline present in the medium. Viable cell concentration (A) and culture viability (B) were determined using a ViCell instrument whilst geometric mean fluorescence (C) was determined using flow cytometry. N = 3 and error bars represent ± one standard deviation.

**Fig. 3:**
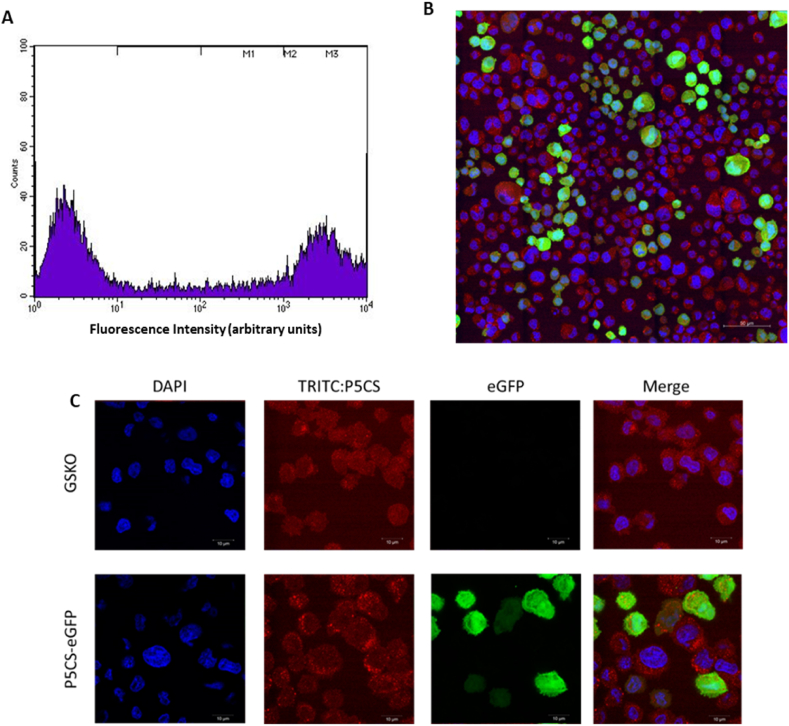
Analysis of cell pools generated through overexpression of P5CS and cultured in L-proline-free medium. Cell cultures were analysed using flow cytometry (A). Fluorescent confocal microscopy was also undertaken where blue = DAPI, red = P5CS:TRITC, green = eGFP (B and C). Figure B shows an image (where all channels are overlaid) of cells generated using the P5CS/proline selection system and Figure C shows a comparison between P5CS/proline generated pools and the GSKO host. (For interpretation of the references to colour in this figure legend, the reader is referred to the Web version of this article.)

**Fig. 4:**
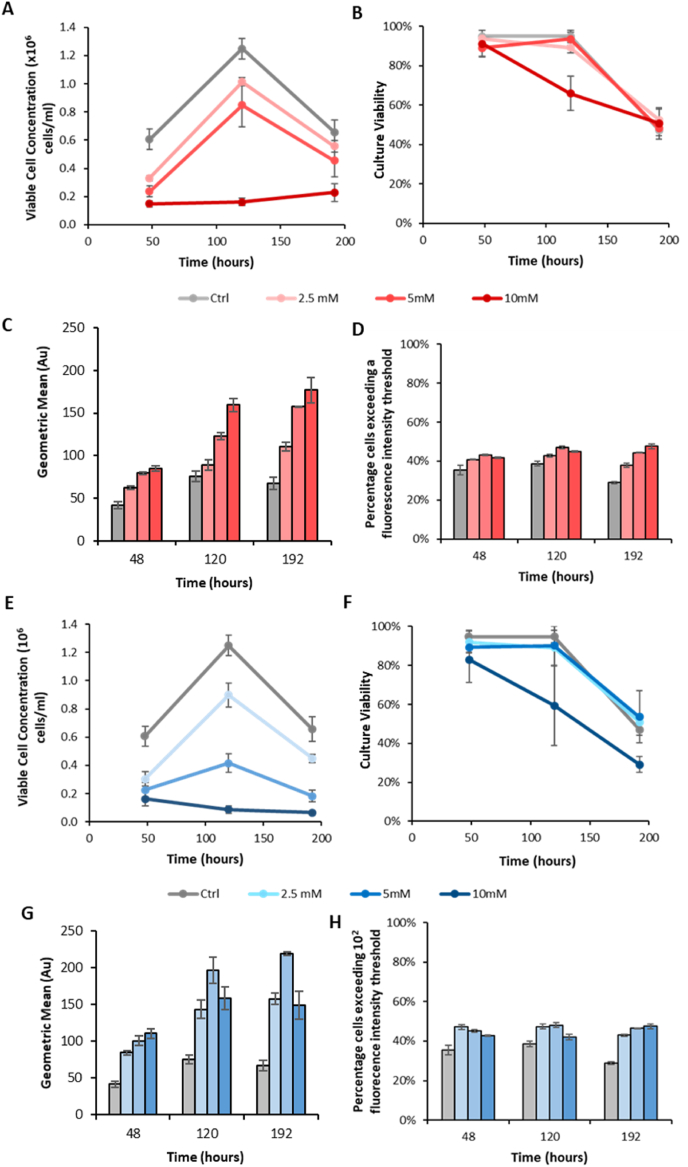
Analysis of cell pools generated using exogenous P5CS expression. Cells selected by culturing transfected cells in L-proline-free Medium A were transferred to media supplemented with known P5CS inhibitors added at a variety of different concentrations and cultured in static 24 well plates for 9 days. Cells cultured in the presence of L-azetidine-2-carboxylic acid (red) were analysed for viable cell concentration (A) and culture viability (B). Cells were also analysed by flow cytometry to determine geometric mean cell fluorescence (C) and the percentage of cells exceeding a predetermined fluorescence threshold (D). In addition cells were cultured in the presence of 3,4- dehydro-L-proline (blue) and analysed for viable cell concentration (E) and culture viability (F). Cells were also analysed using flow cytometry to determine geometric mean cell fluorescence (G) and the percentage of cells exceeding a predetermined fluorescence threshold (H). N = 3 and error bars show ± one standard deviation. (For interpretation of the references to colour in this figure legend, the reader is referred to the Web version of this article.)

### 2.7 Western blot analysis

Total cell protein was extracted from cell pellets by chemical lysis using a lysis buffer supplemented with protease inhibitors as previously described (Roobol et al., 2014). SDS-PAGE was subsequently used to resolve polypeptides from these lysates. Proteins were then transferred to a nitrocellulose membrane and blocked for 30 min in a 5% (w/v) powdered milk solution made up in 0.2% Tween TBS. Anti-SCD1 (*Cell Signalling 2438*), anti-SREBP1 (*Abcam ab3259* or *Fisher* MA511685 SREBP-1 (2A4) Mouse Monoclonal Antibody), anti-P5CS (anti-ALDH18A1; *Sigma* HPA012604) and anti-α-tubulin (a kind gift from Prof K. Gull (Roobol et al., 2009)) primary antibodies were used. Anti-mouse HRP peroxidase (*Sigma A4416*) or anti-rabbit HRP peroxidase (*Sigma A6154*) secondary antibody conjugates were employed as appropriate to detect the relevant polypeptides via chemiluminescence using Hyperfilm ECL reagents (GE Healthcare). Densitometry was carried out using ImageJ software.

### 2.8 Dot blot analysis

Proteins were extracted from cell pellets (section 2.6) and lysate samples drawn through a nitrocellulose membrane using Bio-dot apparatus (*BioRad*). Anti-SREBP1 (*Abcam ab3259*), anti-P5CS (anti-ALDH18A1; HPA012604) or anti-β-actin (*Sigma A5441*) were exposed to the membrane. Anti-mouse HRP peroxidase (Sigma A4416) or anti-rabbit HRP peroxidase (*Sigma A6154*) secondary antibody conjugates were employed to facilitate relevant polypeptide detection via chemiluminescence using Hyperfilm ECL reagents (GE Healthcare).

### 2.9 Genomic DNA extraction and quantitative PCR

Genomic DNA extractions were carried out using a PureLink Genomic DNA Mini Kit (Invitrogen) following the manufacturer's instructions. Quantitative PCR was carried out on 50 ng genomic DNA using Qiagen QuantiFast SYBR Green RT-PCR kits as per instructions provided by the manufacturer. The primers used to quantify exogenous *CgPICR_007864* (for P5CS), *SCD1* and *SREBF1* are shown in Table 1. Forward primers were designed to the different promoters (PGK or SV40) used to drive exogenous expression of *SCD1* or *SREBF1* in order to distinguish between exogenous and endogenous sequences present in the engineered cell genomes.

**Table 1:** Primers used for analysis of genomic *CgPICR_007864* DNA.

Gene Name	Forward Primer (5’ – 3′)	Reverse Primer (5’ – 3′)
*CgPICR_007864* (P5CS)	ccccatggctgactaatttt	gacagctgaaggctggactc
*PGK-SCD1*	cattctgcacgcttcaaaag	tggtggtggtcgtgtaagaa
*PGK-SREBF1*	cattctgcacgcttcaaaag	tgagctgaagcatgtcttgg
*SV40-SREBF1*	ccccatggctgactaatttt	tgagctgaagcatgtcttgg

### 2.10 Confocal microscopy

Coverslips were coated with poly-L-lysine (molecular weight 70,000–150,000 (P4707), Sigma Aldrich, USA) by submerging the coverslip for 15 min in a well of a 24 well plate and subsequently rinsed with water and left to air dry for 1 h. Cells were seeded at 2 × 10^5^ viable cells/ml where 1 ml was placed in each well onto the pre-treated coverslips. These were incubated statically for 1 h at 37 °C and 5% CO_2_ to allow cells to adhere to the coverslip before the medium was aspirated and cells fixed by adding 250 μl 4% (w/v) paraformaldehyde in PBS and subsequently permeabilised using 0.1% (v/v) Triton-X in PBS. Coverslips were transferred to 25 μl drops of anti-P5CS antibody (anti-ALDH18A1; HPA012604) diluted in 3% (w/v) BSA in PBS and incubated overnight. Four washes, each 5 min long, were carried out using 0.1% (v/v) Tween in PBS before exposing to anti-rabbit TRITC antibody (1:100). A further 4 washes were carried out as before and coverslips were transferred to 50 μl drops of DAPI (10 mg/ml) and washed a final 3 times. Finally, cells were mounted using ProLong Gold mounting agent on a glass slide before examination using a Zeiss LSM 880/Elyra/Axio Observer Z1 confocal microscope.

### 2.11 Flow cytometry

Cells were harvested and then the pellets resuspended in PBS. Samples were analysed using a FACScalibur (BD Biosciences) and fluorescence intensity was measured using the FL-1 detector at 473 (Au). The E−1 amplifier was used to collect forward scatter (FSC) and side scatter (SSC) was set to 465 (Au). Thresholds were set such that events recorded below 550 FSC or SSC were not collected as part of the final analysis.

### 2.12 Quantification of secreted antibody production

Recombinant molecule concentrations in cell culture supernatants were determined using an Octet® instrument (ForteBio) with IgG calibrators and protein A biosensors.

## 3 Results

### 3.1 Culture of CHOK1SV GS-KO™ host cell line in L-proline-free medium

We initially tested the CHOK1SV GSKO cell line to confirm the expected proline auxotrophic phenotype of this CHO cell line given its lineage (Kao and Puck, 1967). The CHOK1SV GSKO™ host cell line was grown in batch culture in CD-CHO complete medium (with L-glutamine and L-proline), CD-CHO medium with no L-glutamine or medium with no L-proline. Fig. 1 shows the resulting growth profile of the cell cultures under these conditions over 168 h where cell concentration and culture viability were determined every 24 h. The CHOK1SV GSKO™ host cell line is engineered to knockout glutamine synthetase expression, which has previously been shown as essential for CHO cell growth and survival in the absence of exogenous L-glutamine (Nakamura and Omasa, 2015). As expected, cells grown in the presence of both L-proline and L-glutamine (complete medium) showed a typical batch-culture growth profile over the 168 h of culture, reaching a viable cell concentration of approximately 14 × 10^6^ cells/ml and maintaining a culture viability close to 100% across the culture (Fig. 1). Cells cultured in the absence of L-glutamine did not grow and the viability of these cultures dropped as expected such that by the end culture viability was <10%. As expected, cells cultured in the absence of L-proline showed almost no growth compared to the control, reaching a maximum cell concentration of 0.3 × 10^6^ cells/ml. Culture viability declined albeit at a slower rate compared to L-glutamine-free medium (Fig. 1). Both these observations are in agreement with that expected based on the report of Kao and Puck (1967) who showed that in the absence of proline CHO cells had virtually no growth whilst viability was initially maintained for 2–3 days but then decreased with a half-life of 1.3 days.

As Kao and Puck also showed that spontaneous revertants to proline independence could arise when cells were cultured in the absence of proline, the frequency of occurrence of proline-independent revertants was determined (Section 2.3). The estimated frequency for occurrence of revertants in the absence of proline was 17.4 colony formations per 10^7^ cells plated (data not shown). This compares to that reported by Kao and Puck (1967) of 24 colonies from 1.12 × 10^7^ cells and a spontaneous reversion rate of approximately 1 × 10^−6^. Thus, our findings are in line with those previously reported although we note that the previous studies contained 2% fetal calf serum and were grown in a different medium. This reversion frequency compares to a reversion rate of 0 in the absence of L-glutamine because the introduced *GLUL* deletions in the 2 alleles of the CHOK1SV GSKO™ host cell line are over-lapping and out-of-frame. Thus, as expected given its provenance, CHOK1SV GSKO™ can be considered as a functional proline auxotroph.

### 3.2 Expression of exogenous P5CS restores growth of CHOK1SV GSKO™ cells in L-proline-free medium

We next undertook transient expression studies to determine (1) whether overexpression of P5CS was able to restore the growth attributes of the CHOK1SV GSKO™ host cell in L-proline-free culture media, and (2) the effect upon subsequent transient recombinant protein expression. A vector containing the genes for *CgPICR_007864* (for P5CS) and eGFP was constructed to facilitate these studies. An additional control vector, a negative control vector with the same basic vector structure but lacking both the selection gene and the *eGFP* reporter gene was also used. These vectors are referred to as ‘P5CS’ and ‘control’ respectively. The CHOK1SV GSKO™ host was transfected with the different vectors and cells subsequently cultured in either complete, no L-glutamine or no L-proline media (Fig. 2). Cells transfected with any one of the vectors achieved higher viable cell concentrations in complete medium (containing L-glutamine and L-proline) than their counterparts cultured in the absence of either L-glutamine or L-proline (Fig. 2A).

The resulting data confirmed that exogenous expression of P5CS confers the ability of cells to both survive and grow in L-proline-free medium. Cells transfected with the *CgPICR_007864* containing vector obtained higher viable cell concentrations when cultured without L-proline, than when transfected with the control vector. An increase in culture viability (Fig. 2B) and geometric mean fluorescence (Fig. 2C) was observed when using the *P5CS* vector in combination with the medium lacking L-proline compared to the control vector in L-proline-free medium. The geometric mean was used as it is more robust and less sensitive to outliers than the arithmetic mean. The data suggest that transient overexpression of P5CS is able to restore growth characteristics in cells where no exogenous L-proline is available. Presumably this is enabled through synthesis of additional L-proline (see section 3.7 for confirmation of this). This increase in viable cell concentration, culture viability and fluorescence was only observed with the *CgPICR_007864*/L-proline-free medium combination suggesting that the *CgPICR_007864* gene was expressed to sufficient amounts to restore loss of cellular functions observed when L-proline is removed. Cells cultured in the absence of L-proline achieved a substantially lower maximum viable cell concentration in L-proline-free medium compared to complete medium (Fig. 2A). As previously observed and described in Fig. 1, cells grown in the absence of L-proline had very limited growth, albeit achieving a higher viable cell concentrations than when cultured without L-glutamine (compare No Pro with No Glut, Fig. 2A).

### 3.3 Stable CHOK1SV GSKO™ recombinant protein expressing cell pools can be produced using P5CS as a metabolic selection marker

Following the demonstration that cell growth alongside enhanced survival rates could be achieved in a short, transient setting following introduction of the *CgPICR_007864* gene, we carried out further studies to determine whether the P5CS based selection system was suitable for stable cell pool construction. To achieve this the vector containing the *CgPICR_007864* and *eGFP* genes was linearised and transfected into the Lonza CHOK1SV GSKO™ host cell line. Cells were then left to recover in L-proline-free medium in a static incubator for a total of 14 days before transferring to 20 ml suspension cultures in 125 ml Erlenmeyer flasks. Fig. 3 shows analysis of the resulting cell pools constructed using the P5CS system described. Flow cytometry was used to measure fluorescence arising from eGFP expression in the cell pools; a clear population of cells with high fluorescence was observed (Fig. 3A). Since cells were gated such that hosts expressing no eGFP did not exceed 10^1^ fluorescence intensity, it is apparent that those cells exceeding this threshold had a higher fluorescence intensity due to the expression of eGFP. Analysis of the cell pool constructed using the P5CS selection system showed that some cells within the population did not exceed the fluorescence intensity threshold indicating that they did not express eGFP at detectable levels. Approximately 43% of the cells in the pool population exceeded the pre-set fluorescence intensity threshold.

The cell pool population was also analysed for eGFP and P5CS expression using confocal microscopy imaging (Fig. 3B and C). This analysis again confirmed eGFP expression in the population. The cells expressing eGFP also showed an increased P5CS expression in the pool transfected with the P5CS and eGFP construct compared to the GSKO control host cell pool (Fig. 3C). The analysis of P5CS expression also indicated the localisation of P5CS in CHO cells whereby punctate spots representative of P5CS were observed that are consistent with previous literature showing that P5CS is present in mitochondria (Pérez-Arellano et al., 2010).

### 3.4 Addition of P5CS inhibitors to recombinant stable cell pools constructed with the P5CS/proline selection system enriches the stringency of selection

We next investigated whether the use of inhibitors of the P5CS enzyme could improve the stringency of the selection system in a manner similar to that by which addition of MSX or MTX can improve the selection stringency of the GS and DHFR selection systems respectively. In order to investigate this, inhibitors of P5CS enzyme activity were used to determine if the presence of these would increase the stringency of the selection system (i.e. level of metabolic marker expression required for a transfected cell to survive in the selective environment resulting in death of both non-transfected cells and cells with low level expression of the selectable marker). In theory, by downregulating P5CS activity and therefore proline synthesis only those cells with higher exogenous P5CS expression would be able to survive, grow and proliferate in L-proline-free medium. Fig. 4 shows the analysis of the cell pool constructed above (Fig. 3) and grown in the presence of a range of concentrations of the P5CS inhibitors L-azetidine-2-carboxylic acid (Fig. 4A–D) or 3,4-dehydro-L-proline (Fig. 4E–H) added into L-proline-free medium A at inoculation of the cultures. We note that whilst L-azetidine-2-carboxylic acid may be misincorporated into polypeptides in place of proline (Samardzic and Rodgers, 2019) which is undesirable and potentially toxic, to our knowledge 3,4-dehydro-L-proline is not highly toxic which is a further potential benefit of using this inhibitor.

Determination of viable cell concentrations and culture viability plus analysis by flow cytometry fluorescence were carried out at 48, 120 and 192 h post supplementation whereby cultures were initially seeded at 0.2 × 10^6^ viable cells/ml in static 24 well plates. The resulting data show that both L-azetidine-2-carboxylic acid and 3,4-dehydro-L-proline reduced the estimate of maximum viable cell concentration (value at 120 h), reduced overall culture viability but increased the percentage of cells expressing eGFP and the overall geometric mean fluorescence (Fig. 4). This suggests that inhibition of P5CS activity was able to increase the stringency of the P5CS selection system over a 192 h time period. Furthermore, the effect of the chosen inhibitors was concentration dependent in that higher concentrations of inhibitor generally increased the geometric mean fluorescence of the culture the most (Fig. 4D and H) and reduced viable cell concentration of the pool the most (Fig. 4A and E). Whilst geometric mean fluorescence increased with increasing L-azetidine-2-carboxylic acid concentration in all cases, for 3,4-dehydro-L-proline the geometric mean fluorescence of the population increased when supplemented with 2.5 or 5 mM but the use of 10 mM resulted in a decrease in mean fluorescence when compared to 5 mM (Fig. 4H). In summary, 10 mM L-azetidine-2-carboxylic acid and 5 mM 3,4-dehydro-L-proline were the most effective concentrations evaluated for improving P5CS selection system stringency over the time period analysed.

### 3.5 Use of P5CS/proline selection in engineering of lipid biosynthesis in the CHOK1SV GSKO™ host cell line

Following the successful demonstration that the P5CS/proline system could be used to select for cells expressing a model protein, we employed the system to engineer lipid metabolism in the CHOK1SV GSKO™ host cell line. We used the system to select for cells stably overexpressing two different lipid metabolism modifying (LMM) genes, *SCD1* and *SREBF1*. We have previously shown that overexpression of these genes in a CHO cell host can change lipid metabolism, expand the ER of CHO cells and enhance productivity of biotherapeutic protein molecules such as mAbs (Budge et al., 2019). We therefore attempted to engineer CHOK1SV GSKO™ cell pools to overexpress these two LMM by transfecting vectors containing the *CgPICR_007864* (for P5CS) gene, to facilitate selection, and either the *SCD1* or *SREBF1* genes (where expression was driven by different promoters compared to the *CgPICR_007864* gene) into the host and subsequently allowing the cells to recover in L-proline-free CD-CHO medium. The transfected cells were plated out to create minipools (i.e. transfected cells were distributed across 96 well plates such that each well contained 10,000 transfected cells). Since the optimal expression level of the LMM genes was unknown, inhibitors to improve stringency were not employed during this construction process thus potentially allowing cells with a range of P5CS and LMM expression levels to recover to facilitate evaluation of lower and higher expressing cells alike.

**Fig. 5:**
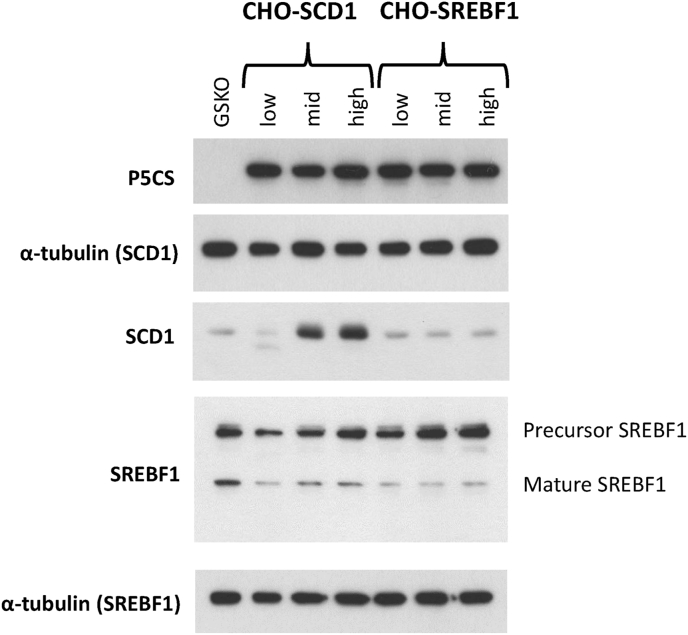
Western blot analysis of CHOK1SV GSKO™ cells engineered to overexpress either SCD1 or SREBF1 using the P5CS/proline selection system. The cells were ranked for levels of LMM expression and pooled in order to generate low, mid and high overexpressing pools. Western blots have been appropriately exposed to antibodies to indicate bands associated with P5CS, SCD1 and SREBF1 where α-tubulin was used as a loading control (since SCD1 and SREBF1 were probed on different blots, separate loading controls from each blot have been included as indicated).

The resulting minipools generated were analysed via dot blot to determine the relative levels of LMM protein expression (Supplementary Figure 1 shows examples of dot blots obtained). The minipools were then ranked based on the relative amounts of the LMM protein expressed (within the range generated from specific constructs) and 20 of those with similar expression levels as determined by dot blot combined to generate a ‘low’, ‘mid’ and ‘high’ expressing pool. Fig. 5 shows western blot analysis of cell lysates generated from cells from each of these pools. Whilst the CHOK1SV GSKO™ control host showed no detectable levels of P5CS protein by western blot, the pools all showed expression of the P5CS protein confirming that over-expression had been achieved in the arising cell pools (Fig. 5). Furthermore, the western blot analysis confirmed that the application of the P5CS selection system had resulted in the isolation of pools overexpressing the target LMM molecules as shown by increased SCD1 and precursor SREBF1 protein expression levels in the high pools (Fig. 5). This provides clear evidence that the P5CS system can be used in CHO cells without the use of inhibitors to isolate cells expressing target exogenous genes that reside within the same genetic construct.

### 3.6 LMM engineered CHOK1SV GSKO™ cell pools generated using the P5CS based selection system show stability for at least 100 generations

**Fig. 6:**
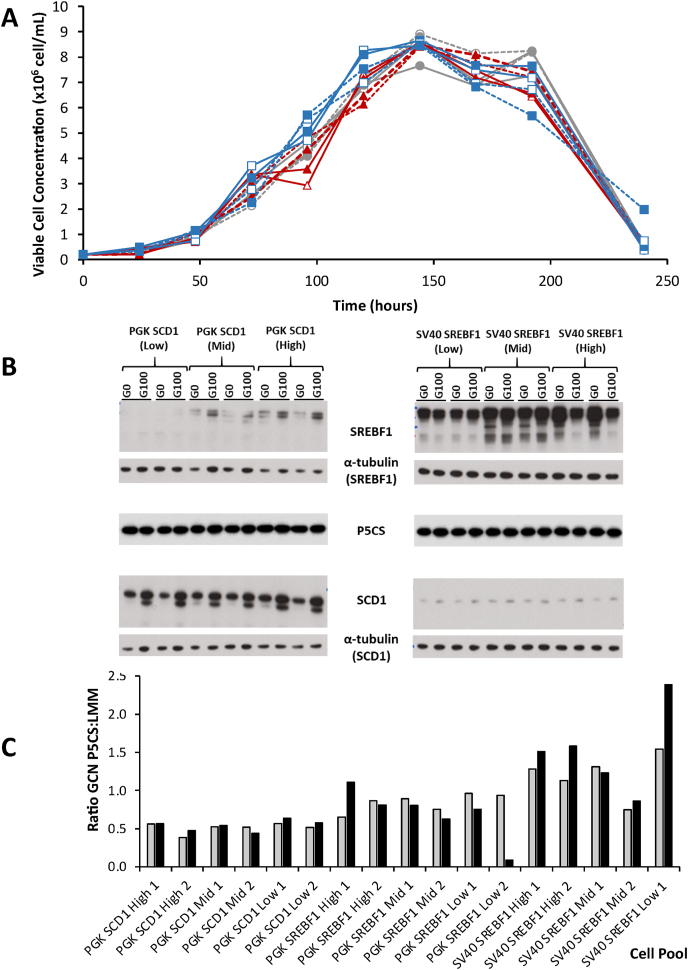
Assessment of the long term stability of CHOK1SV GSKO™ cells engineered to overexpress the *SCD1* and *SREBF1* LMM genes using the PSCS/proline selection system. (A) Engineered cells were maintained for 100 generations with cells passaged every 3–4 days in L-proline free medium and batch culture experiments were run to compare cells derived from the 1st (G0) and 100th generation (G100). The growth profiles were determined by measuring the viable cell concentration of cell pools across batch culture. Control cell pools were generated using constructs containing the P5CS selection marker with no additional gene (fx). Cell pools overexpressing SCD1 were constructed with a vector containing both the *P5CS* selection marker and *SCD1* gene where expression was driven by a PGK promoter and pools considered either relatively high (fx) or low (fx) overexpression levels. Two independeht pools were monitored and these are distinguished by either an open marker or file d marker. Solid lines show *“*GO*”* and broken lines show *“*G100*″* cultures. (B) Western blot analysis of cell lysate samples harvested from cells at GO and G100 for P5CS, SCD1 and SREBF1 protein expression where α-tubulin was used as a loading control. As SCD1 and SREBF1 were probed for on different blots, separate loading controls from each blot have been included as indicated. (C) Quantitative PCR was carried out to compare gene copy numbers of cell pools from GO (grey bars) or G100 (black bars). The ratio of P5CS:LMM gene copies (GCN - gene copy number) was determined from data obtained from each timepoint. Samples 1 and 2 refer to biological repeats and promotors, LMM and relative overexpression levels are labelled accordingly.

Generating recombinant engineered cell pools and lines able to sustain a consistent and high expression of exogenous target genes over long periods of time is an ongoing challenge for industry (Dahodwala and Lee, 2019). Selection systems employed for generating engineered and recombinant CHO cell lines must be evaluated for their capacity to maintain prolonged stability of exogenous gene expression. With this in mind, we assessed the long-term stability of the P5CS/proline based system employed here in terms of both the selectable marker P5CS and the exogenous SCD1 and SREBF1 LMM genes introduced using this selection system. The engineered cell pools were maintained in L-proline-free CD-CHO medium (Gibco) over 100 generations where cells were harvested and lysed at 0 and 100 generations (generation or population doubling number (n) calculated as n = (log cell number of subculture - log seeding cell number of subculture)/log (2) + x where x = generation number of culture used for seeding). Fig. 6A shows batch culture growth profiles of SCD1 engineered cells from generation 0 and 100 that were run simultaneously after revival from cryostorage. Supplementary Table 1 reports the IVC, maximum viable cell concentration, growth rate and doubling time for all SCD1 and SREBF1 pools. Importantly, the batch culture growth profiles of cells after 100 generations showed no marked change compared to those from generation 0 (Supplementary Table 1), indicating that the ability to grow in L-proline-free medium was consistent over 100 generations. Further evidence of the maintained growth profiles is illustrated in Supplementary Figure 2 which reports addition cell pool G0 and G100 growth profiles.

Analysis was also carried out on protein lysates harvested at generation 0 and 100 (G0 and G100) to compare expression levels of SCD1 or SREBF1 between these generations. Fig. 6B shows western blot analysis of P5CS, SCD1 and SREBF1 expression where α-tubulin was used as a loading control. The relative levels of the P5CS selection marker are similar between the respective G0 and G100 samples and between different cell pools, indicating the stability of P5CS expression over time. This was also the case for the low and mid-SREBF1 pools (Fig. 6B). For the highest expressing SREBF1 pools (SV40 SREBF1 High) there was a change in banding pattern between G0 and G100 samples (Fig. 6B), consistent with previous studies, suggesting that high levels of SREBF1 are not maintained due to lipotoxicity associated with overproduction of cellular lipids brought about by upregulation of SREBF1 (Kliewer et al., 1997; van Herpen and Schrauwen-Hinderling, 2008). On western blots for the SCD1 protein for G100 samples a lower band was observed in addition to the single higher band observed in G0 samples (Fig. 6B). It is likely that the double SCD1 band observed in G100 samples of SCD1 overexpressing pools was due to inherent lipid metabolism regulatory processes which have been reported to occur later in subculture (Heinemann and Ozols, 1998). In this case, samples were taken during routine passaging which occurred 3 days after seeding for G0 samples and 4 days after seeding for G100 samples.

In addition to analysis at the protein level, qPCR was carried out on genomic DNA isolated from G0 and G100 cultures samples taken at the same time as the protein lysate samples. Fig. 6C shows the ratio of relative P5CS:LMM gene copies, where LMM refers either to *SCD1* or *SREBF1* depending on which gene was being overexpressed in the respective pool. These data show that the ratio of the selection marker (P5CS) and the recombinant target (LMM) was consistent with increasing cell age for almost all of the cell pools evaluated. It is notable that *PGK SREBF1 High 1*, *SV40 SREBF1 High 2* and *SV40 SREBF1 Low 1* samples suggest either a decrease in P5CS or increase in LMM copies between G0 and G100. Since the protein levels of P5CS were remarkably consistent, this is consistent with this increase in ratio being due to a reduction in SREBF1. Conversely, the *PGK SREBF1 Low 2* sample showed results consistent with an upregulation of the SREBF1. Collectively, the data described in this section show that the P5CS/proline system has the capacity to select for stably expressing P5CS cells that maintain their growth phenotype and to maintain cell pools overexpressing the target LMM genes over 100 generations. We note these experiments were conducted using minipools and thus will be more susceptible to variation than clonal cell lines and selection pressure was only maintained by removing L-proline from medium without the addition of inhibitors.

### 3.7 Generation and analysis of monoclonal SCD1 engineered CHOK1SV GSKO™ cell lines from P5CS selected pools

**Fig. 7:**
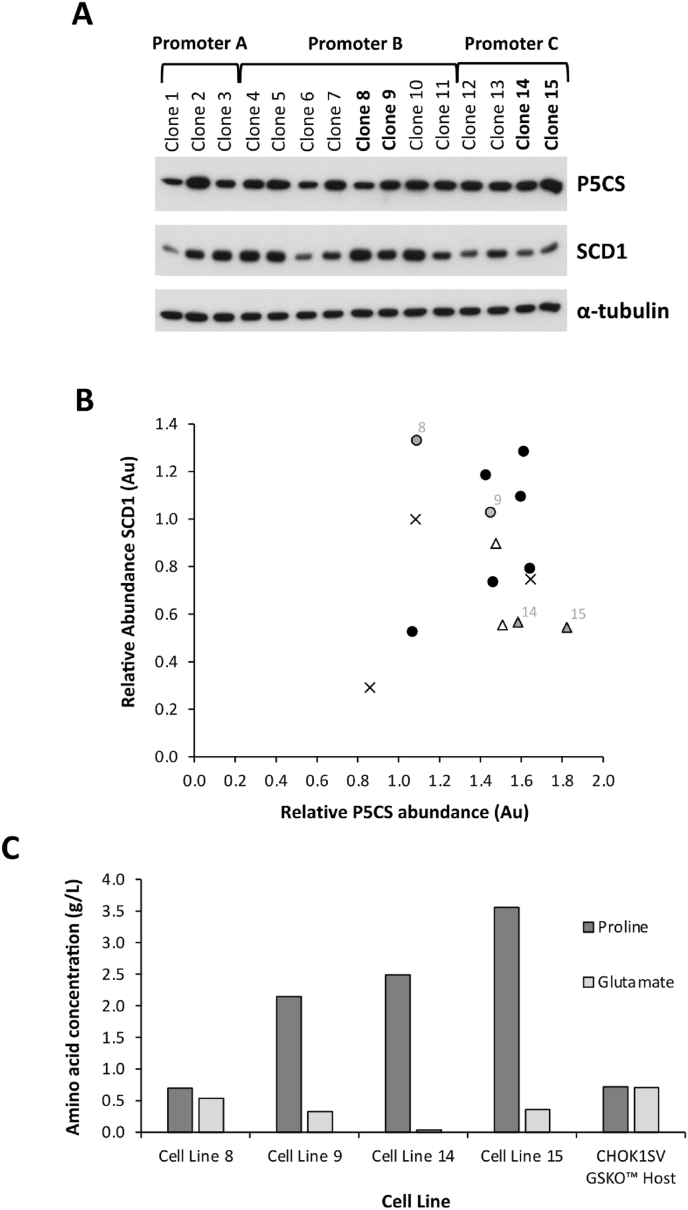
Analysis of isolated SCD1 engineered cell lines constructed using the P5CS selection system. Western blot analysis was used to determine the relative abundance of P5CS and SCD1 present in cell lysates of isolated cell lines engineered to overexpress SCD1 where expression was driven by different promoters and α-tubulin was used as a loading control (A). Densitometry analysis was carried out on western blots to quantify the relative amounts of SCD1 and P5CS where both were normalised to the appropriate α-tubulin band. The relative abundance of SCD1 and P5CS were plotted against each other where an “x" shows samples where promoter A was used to drive SCD1 expression, "•" shows where promoter B has been used and “fx” shows where promoter C has been used. The cell lines which were selected for further evaluation are identified using a grey shape and labelled with the appropriate cell line number (B). The concentrations of proline and glutamate in spent culture media were recorded after 15 days of culture with the specified clones (C).

We have previously shown, using an alternative antibiotic based selection marker, that genetic engineering to modify lipid metabolism by over-expression of SCD1 or SREBF1 can be employed in CHO cells to increase recombinant product yields (Budge et al., 2019, 2020). In the present study, the P5CS/proline selection system was used to genetically engineer the CHOK1SV GSKO™ host cell line through overexpression of the *SCD1* gene as an exemplar of the system as our previous study showed SCD1 over-expression tended to give greater enhancement of recombinant protein production than SREBF1. Engineered pools were isolated that overexpress *SCD1* to different levels in order to find the SCD1 overexpression level that maximises productivity. We initially assessed minipools of SCD1 or SREBF1 engineered CHOK1SV GSKO™ cells for their ability to transiently express a model Fc-fusion protein and what the authors consider a difficult to express mAb in a 96 deep well plate format. From these data, the top 15 minipools, in terms of the Fc-fusion protein and mAb expression were stably transfected, using GS selection, with the two test molecules and then the productivity of the resulting pools assessed in an Ambr15® fed-batch experiment (data not shown). Cells from the 5 minipools across the original SCD1 and SREBF1 P5CS engineered hosts that gave the highest mAb and Fc-fusion protein titre were then isolated by limited dilution cloning. Thus, clonal host cell lines expressing SCD1 or SREBF1, but not the model proteins, were obtained from the cell pools that gave the highest secreted product concentrations (data not shown). The resulting cell lines were then again assessed for their ability to transiently express model recombinant proteins in a 96 DWP. The top 15 clonal cell lines were then progressed for further analysis. Fig. 7A shows western blot analysis carried out on these clones to evaluate the relative amounts of P5CS and SCD1 expressed using α-tubulin as a loading control. As expected, the levels of P5CS and SCD1 vary between the clones: Fig. 7B shows the relationship between the relative levels of SCD1 and P5CS for each clone as determined by densitometry. As these were cell lines with different promoters driving SCD1 expression, P5CS expression was always driven by the same promoter and there was a narrow range of P5CS and SCD1 expression in the selected cell lines (Fig. 7B) it is not surprising that there was no evidence for a correlation between P5CS and SCD1 protein expression (Pearson correlation coefficient = 0.10).

In Fig. 7B the densitometry analysis of western blot bands of cell lines 8, 9, 14 and 15 is highlighted. These were the best performing of the cell lines evaluated in terms of transient recombinant protein expression yields (data not shown). Further analysis of these clones was therefore carried out to confirm these cells were able to synthesise their own proline requirements by measuring the proline concentrations in spent medium after 15 days of fed-batch culture in an Ambr15 instrument using L-proline-free Medium A and a Lonza proprietary feeding regime (Fig. 7C). The results show that all of the cell lines were able to synthesise proline and that for 3 of the 4 SCD1 engineered cell lines the concentration of proline detected in the spent medium was approximately 4-6-fold higher than in the spent medium of the CHOK1SV GSKO™ host cultured using medium containing the standard amount of L-proline. The other SCD1 engineered host had a similar amount of proline in the spent medium compared to that in the control host. These data confirm that the SCD1 engineered cells were able to produce endogenous proline, presumably due to the presence of the *P5CS* gene which facilitates cell survival and growth. Indeed, 3 of the cell lines appear to produce much more proline than required by the cell as evidenced by the high concentrations observed in the spent media. The levels of glutamate, a precursor in the biosynthesis of proline, were also determined and are reported in Fig. 7C. All the SCD1 engineered clones generated using the P5CS selection system had lower levels of glutamate in the spent medium compared to the control host, suggesting that the cells utilise glutamate to synthesise proline via the P5CS selection system. Thus, where higher levels of proline were observed, lower levels of glutamate were detected.

### 3.8 The GS selection system can be used in conjunction with the P5CS proline based selection system to generate cells engineered to overexpress SCD1 and to produce a model difficult to express biotherapeutic monoclonal antibody

**Fig. 8:**
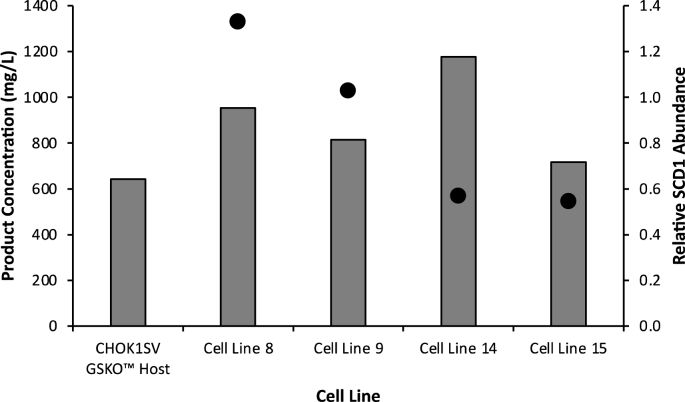
Difficult to express (DTE) monoclonal antibody product concentrations from cell pools generated using the GS expression system where engineered clones overexpressing SCD1 (established using the P5CS selection system) were used as the host for cell pool construction as shown on the x axis. Each bar shows the product concentration values from a cell pool construction process where the top 10 yielding minipools were pooled and an established fed-batch production protocol in an Ambr15® used to assess product yields from the resulting combined pool. The secondary axis shows the relative abundance of SCD1 as determined using data in Fig. 7 where the data points are denoted by circles in line with the appropriate titre data for each cell line. The CHOK1SV GSKO™ host and GS selection for the isolation of antibody expressing pools was used to compare against the performance of SCD1 engineered host cells selected using the P5CS system.

It is critical that any selection strategies used to engineer CHO cells to introduce genes that will improve the desired characteristics of the host cell line is compatible with selection strategies used to subsequently select for cells producing a recombinant product in the engineered host. As such, an established commercially relevant cell line construction workflow at Lonza which utilises the GS selection system was applied to the four SCD1 engineered cell lines 8, 9, 14 and 15 (Section 3.7) to generate cell pools expressing a clinically relevant model recombinant monoclonal antibody product considered difficult to express (DTE) by the authors. The top 10 expressing minipools for each cell line, as determined by Protein A Octet analysis at the 96 DWP scale, were then pooled together. These new minipools were subsequently grown up into shake flasks and then grown under fed-batch culture conditions in an Ambr®15 bioreactor system in L-proline-free Medium A and the concentration of secreted antibody in the cell culture supernatant at the end of culture determined by Protein A Octet analysis. Fig. 8 and Supplementary Table 2 report the product concentrations obtained from the pools originating from the different host cell lines. Importantly, the data show that the GS selection system can be used subsequent to, and on top of, the P5CS selection system to generate recombinant monoclonal antibody secreting cell pools that produce as well, if not better, than the original non-engineered cell line (Fig. 8). The cell specific productivity (Qp) was increased in all SCD1 engineered pools compared to the control CHOK1SV GSKO™ host, further confirming that over-expression of SCD1 can enhance cell specific and overall productivity from CHO cells and that this can be achieved using P5CS selection. Thus, overall these data show that P5CS can be used to engineer CHO cells without interfering with subsequent cell line construction using the established GS selection system. Increased product yields were also achieved from LMM engineered cells which is consistent with previous studies showing that overexpression of SCD1 can improve recombinant antibody concentrations from CHO cells (Budge et al., 2019).

## 4 Discussion

As outlined in the introduction, selection marker technologies are routinely employed to generate recombinant mammalian cell lines producing a range of biotherapeutic protein molecules of interest. After a lack of developments over the last several decades there has been an upsurge of interest with a number of groups reporting the recent development of alternatives to GS and DHFR metabolic selection systems for use in CHO cells (Kaufman and Sharp, 1982a; Jossé et al., 2018; Cockett et al., 1990; Birch et al., 2008). Additional selection systems also allow for the engineering and maintenance of host cell systems to improve, or introduce new, cellular attributes whilst leaving, for example, the traditional GS and DHFR systems available for the subsequent introduction of a target biopharmaceutical. Here, the ability of the P5CS/proline system to be employed for cell line engineering has also been demonstrated, being used to select for cells overexpressing the lipid metabolism modifying genes *SCD1* and *SREBF1*. Further, we have previously described how the SCD1 enzyme, that converts saturated fatty acids to monounsaturated fatty acids, can be overexpressed to enhance recombinant protein productivity (Budge et al., 2019). We were able to replicate this result using the P5CS selection system to engineer the CHOK1SV GSKO™ host cell line and subsequently use the engineered host to generate cell pools using the established GS system expressing industrially relevant amounts of a difficult to express monoclonal antibody. The compatibility of these systems together has great potential in cell line engineering but may also be utilised for production of materials where it is necessary to express multiple genes. Additionally, the P5CS engineering of the cells removes the need to provide L-proline in the cell culture medium and therefore has implications for media development and bioprocessing. Such an ability to engineer host cells also opens the opportunity to remove medium components that can be problematic during bioprocessing or change metabolism to prevent the accumulation of toxic growth inhibitors as demonstrated by others who engineered CHO cell phenylalanine-tyrosine and branched chain amino acid biosynthesis to prevent the build-up of such growth inhibiting compounds so that it was not necessary to add tyrosine to the culture media (Mulukutla et al., 2019).

The studies described here outline development and validation of a selection system centred around proline synthesis, showing its successful implementation and the stability of the system over long-term culture to provide a selective advantage (i.e. the ability to grow in L-proline-free medium) to CHO cells expressing recombinant genes of interest. The fundamental basis of the selection system relies on the overexpression of the gene for P5CS combined with the use of L-proline-free culture medium to enrich a cell population expressing a desirable second (or additional) exogenous gene located on the same construct as the gene for P5CS. Whilst we have shown that this system can be successfully employed simply by removing exogenous L-proline from the culture medium, we have also successfully demonstrated the utility of inhibitors of the P5CS enzyme to improve selection stringency. A crucial finding in this study is that the cells selected using this system exhibit very similar growth profiles in comparison to the parental host (cultured with exogenous L-proline) and are able to proliferate in the absence of L-proline over more than 100 generations. This allows for sustained selection pressure in culture simply by removing exogenous L-proline and means that the selection method is compatible with existing processes beyond cell line construction.

Finally, it is essential that a selection system designed to generate high producing recombinant cell lines is able to sustain stable and high production levels over time. Knockout of endogenous GS in CHO cell hosts has proved successful in enhancing selection stringency and this increases the likelihood of selecting a superior clone (Dahodwala and Lee, 2019; Jun et al., 2006; Chusainow et al., 2009). We have shown that overexpression of a transgene in cell pools generated from P5CS engineered hosts were maintained across 100 generations in the majority of pools investigated. Whilst expression levels in cell pools may be subject to fluctuation over time due to their heterogeneous nature, monoclonal cell lines are likely to exhibit increased stability. We observed low P5CS expression amounts in native CHO host cells, in agreement with a previous study by Sun et al. that also demonstrated that knockout of the P5CS gene can improve the stringency of a proline based selection system (Sun et al., 2020). We have shown that enhanced stringency can be achieved without knockout when supplementing culture medium with appropriate inhibitors of the P5CS enzyme (L-azetidine-2-carboxylic acid or 3-4-dehydro-L-proline) and therefore use of such inhibitors can improve stringency without the need for P5CS knockout. A combination of the P5CS knockout strategy and addition of the inhibitors outlined here could provide the selection system with additional stringency.

Collectively, the data presented here demonstrate that the P5CS proline selection system can be used for the isolation of cells that grow in the absence of exogenous L-proline with a growth profile similar to that of the original host. Further the system can be used to introduce exogenous gene function to CHO cells that is stable under continued selection based upon the absence of L-proline in the culture media, and that such engineered host cells can subsequently be used to generate stably expressing biotherapeutic protein cell pools based upon GS selection technology at industrially relevant levels. In this aspect our study differs from that of Sun et al. who utilised the P5CS system to directly select for biotherapeutic mAb production whereas we have combined two metabolic selection systems to (a) firstly utilise the P5CS system to engineer the proline AND lipid metabolism of the cell and then (b) use the GS selection system to select for high biotherapeutic expressing cells. Although we observed no obvious differences in product quality between biotherapeutic proteins produced from the original CHOK1SV GSKO™ host or the SCD1 engineered hosts selected via the P5CS system, whether the engineered systems impact such critical quality attributes could be evaluated further in the future.

In summary, we have successfully designed and implemented a P5CS/proline based system for the selection of recombinant CHO cells under industrially relevant conditions. The system is effective in its current format and inhibitors have been identified which have the potential to improve selection stringency further alongside introduction of additional exogenous genes using alternative, existing selection strategies such as the GS selection technology. The development of such systems opens up new opportunities in the engineering biology field to engineer CHO cells further, and impact bioprocesses, to enhance the ability of such expression systems to produce current, new and novel format biopharmaceutical candidates at higher yield and quality than currently achievable.

## Author statement

James D Budge: Conceptualization, experimental planning, investigation, visualization, writing original draft, writing - review and editing.

Joanne Roobol: Investigation, experimental planning, visualization, writing - review and editing.

Gurdeep Singh: Investigation, experimental planning, visualization, writing - review and editing.

Théo Mozzanino: Investigation, experimental planning, writing - review and editing.

Tanya J Knight: Investigation, experimental planning, project administration.

Jane Povey: Investigation, experimental planning, writing - review and editing.

Andrew Dean: Investigation, experimental planning, visualization, writing - review and editing.

Sarah J Turner: Supervision, experimental planning, writing - review and editing.

Colin M Jaques: Conceptualization, supervision, experimental planning, writing - review and editing.

Robert J Young: Conceptualization, experimental planning, writing - review and editing.

Andrew J Racher: Conceptualization, supervision, funding acquisition, experimental planning, writing - review and editing.

C Mark Smales: Conceptualization, supervision, funding acquisition, experimental planning, writing - review and editing.

## Declaration of competing interest

AD, SJT, CMJ, GS, and AJR are employed by Lonza Biologics, who developed and license the GS Gene Expression System®. Lonza is the assigned owner of, and CMS, JDB, TJK, and RJY are named inventors on, the filed patent ‘Modulation of lipid metabolism for protein production’, patent number WO2017191165A1. Lonza is assigned owner of, and JDB, CMJ, AJR, JR, GS, CMS and RJY named inventors on, the filed patent ‘Methods of cell selection and modifying cell metabolism’ patent number WO2019152876AA2. The authors declare no other financial or commercial conflict of interest.
