# Supplementary material for: A proline metabolism selection system and its application to the engineering of lipid biosynthesis in Chinese hamster ovary cells
Source: Metab Eng Commun. 2021 Jul 27;13:e00179. doi: 10.1016/j.mec.2021.e00179 (PMC8346673; doi:10.1016/j.mec.2021.e00179)
Supplement: Multimedia component 1 [file mmc1.pdf]

Supplementary Table 1: Growth parameters for duplicate batch cultures (designated a and b) of P5CS selected lipid engineered CHOK1SV GSKO™ pools at generation 0 (T0) and generation 100 (T100). Stock of cryopreserved pools at T0 and T100 cultured in the absence of proline with passaging every 3 or 4 days were revived and then batch cultured in Medium A in the absence of exogenous L-proline. Cell culture parameters (culture viability, viable cell number, total cell number) were determined every 24 h. Cell specific growth rate and doubling time were determined over the first 96 h of culture during exponential growth phase.

Cell Pool	IVC ($x10^6$ $cell.ml^{-1}.h$)	Maximum Viable Cell Conc ($x\ 10^6$ ml)	Cell specific growth rate (μ)	Doubling Time (Td, h)
P5CS Control T0 a	1063	8.63	0.034	20.4
P5CS Control T0 b	979	7.65	0.035	19.6
P5CS Control T100 a	1080	8.91	0.033	21.3
P5CS Control T100 b	1034	8.81	0.035	20.0
PGK SCD1 High T0 a	986	8.56	0.037	18.6
PGK SCD1 High T0 b	993	8.60	0.036	19.5
PGK SCD1 High T100 a	1026	8.41	0.035	20.1
PGK SCD1 High T100 b	1031	8.46	0.036	19.4
PGK SCD1 low T0 a	1080	8.45	0.036	19.3
PGK SCD1 low T0 b	1113	8.66	0.035	19.1
PGK SCD1 low T100 a	1034	8.55	0.036	19.1
PGK SCD1 low T100 b	1029	8.46	0.036	19.3
PGK SCD1 mid T0 a	1097	8.78	0.036	19.1
PGK SCD1 mid T0 b	1016	7.83	0.036	19.3
PGK SCD1 mid T100 a	977	8.80	0.036	19.4
PGK SCD1 mid T100 b	930	7.23	0.032	21.9
PGK SREBF1 High T0 a	1012	7.28	0.033	21.3
PGK SREBF1 High T0 b	1079	8.17	0.033	21.0
PGK SREBF1 High T100 a	1014	7.00	0.033	21.0
PGK SREBF1 High T100 b	1039	8.10	0.034	20.6
PGK SREBF1 low T0 a	1166	9.19	0.033	20.8
PGK SREBF1 low T0 b	1053	8.63	0.037	18.7
PGK SREBF1low T100 a	1178	8.28	0.034	20.6
PGK SREBF1 low T100 b	1150	7.93	0.037	18.8
PGK SREBF1 mid T0 a	1107	9.00	0.036	19.1
PGK SREBF1 mid T0 b	985	8.41	0.032	21.7
PGK SREBF1 mid T100 a	1278	8.84	0.034	20.3
PGK SREBF1 mid T100 b	1112	7.97	0.036	19.4
SV40 SREBF1 High T0 a	884	8.28	0.037	18.9
SV40 SREBF1 High T0 b	1071	8.76	0.036	19.3
SV40 SREBF1 High T100 a	968	8.28	0.032	21.5
SV40 SREBF1 High T100 b	971	8.45	0.034	20.1
SV40 SREBF1 low T0 a	915	7.22	0.034	20.6
SV40 SREBF1 low T0 b	953	7.32	0.037	19.0
SV40 SREBF1low T100 a	1069	9.15	0.033	21.2
SV40 SREBF1 low T100 b	980	7.90	0.033	20.9
SV40 SREBF1 mid T0 a	871	7.29	0.030	22.9
SV40 SREBF1 mid T0 b	963	8.28	0.033	21.0
SV40 SREBF1 mid T100 a	1066	8.13	0.036	19.0
SV40 SREBF1 mid T100 b	933	6.81	0.031	22.1

Supplementary Table 2. Productivity and growth characteristics of model difficult to express recombinant mAb producing CHOK1SV GSKO™ cell pools generated using GS selection and different P5CS SCD1 engineering host cell lines or the control CHOK!SV GSKO™ host. Cell pools were cultured in an Ambr®15 instrument in Medium A in the absence of exogenous L-proline using a Lonza Biologics proprietary feeding regime for 15 days.

**Host Cell Line**	**Day 6**			**Day 15**			
	IVC (10^6 cells.h/mL)	Qp (pg/cell/h)	Titre (mg/mL)	IVC (10^6 cells.h/mL)	Qp (pg/cell/h)	Titre (mg/mL)	Culture [Proline] (g/L)
CHOK1SV GSKO™ Host	851	0.108	92	4743	0.136	643	0.72
Cell Line 8	497	0.115	57	4609	0.207	954	0.70
Cell Line 9	682	0.104	71	3413	0.239	815	2.15
Cell Line 14	861	0.183	158	4770	0.247	1177	2.49
Cell Line 15	903	0.164	148	3582	0.200	716	3.56

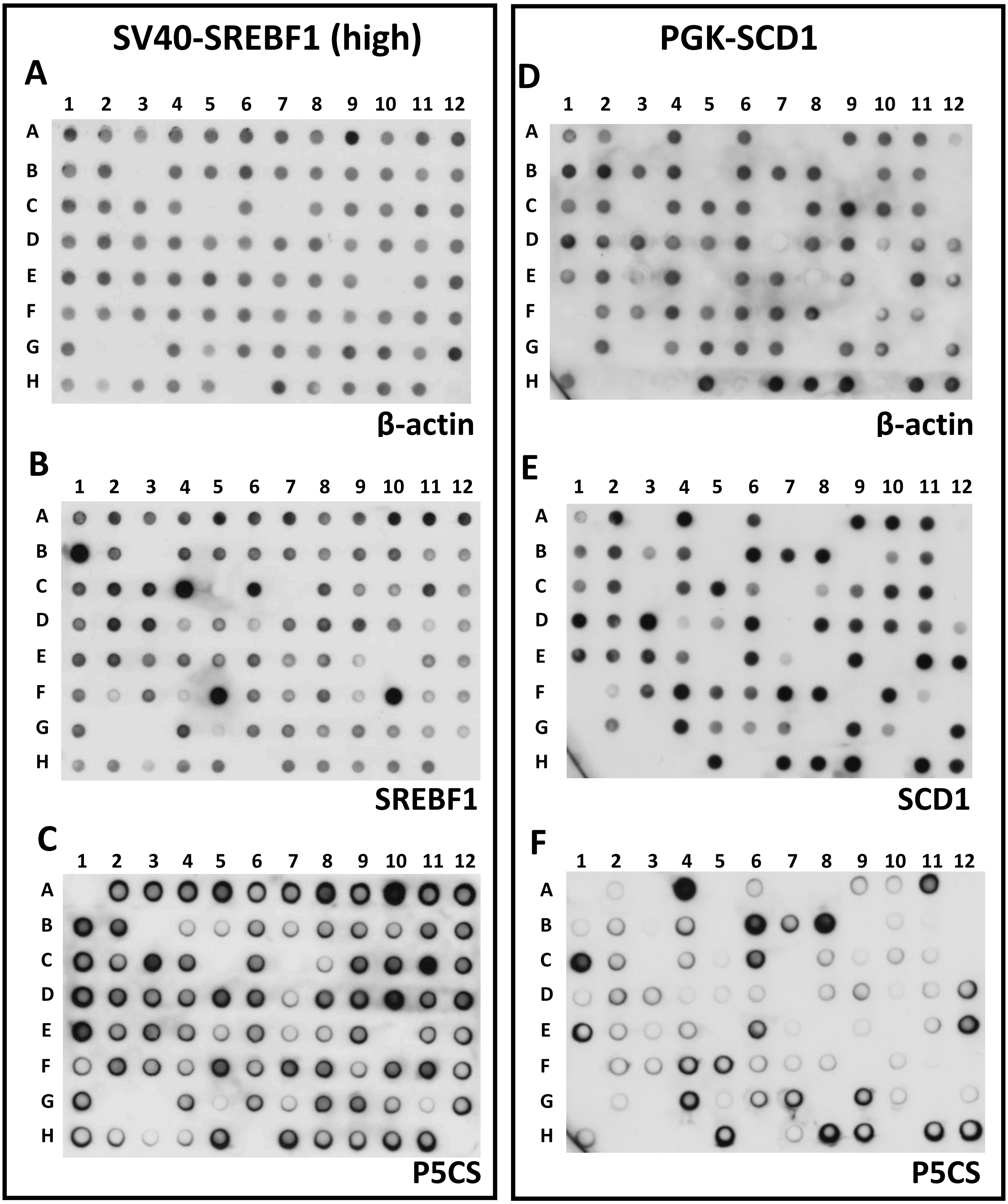

Supplementary Figure 1: Relative levels of selected proteins extracted from engineered minipools which were constructed using Lonza's CHOK1SV GSKO™ cell line using vectors containing the gene for P5CS and either *SREBF1* (expression driven from the SV40 promoter; figures A-C) or *SCD1* (expression driven from the PGK promoter; figures D-F). Dot blots were carried out and appropriate antibodies were used to probe for either β-actin (A & D), which was used as a loading control, P5CS (C & F) and either SREBF1 (B) or SCD1 (E) dependent on which of these was being overexpressed in the modified pools. In each dot blot, lysate harvested from the unmodified CHOK1SV GSKO ™ host line was loaded in well A1 as the positive control. These are examples of dot blots which were used, in part, to select for minipools expressing the lipid modifying genes at 'high', 'mid' and 'low' amounts.

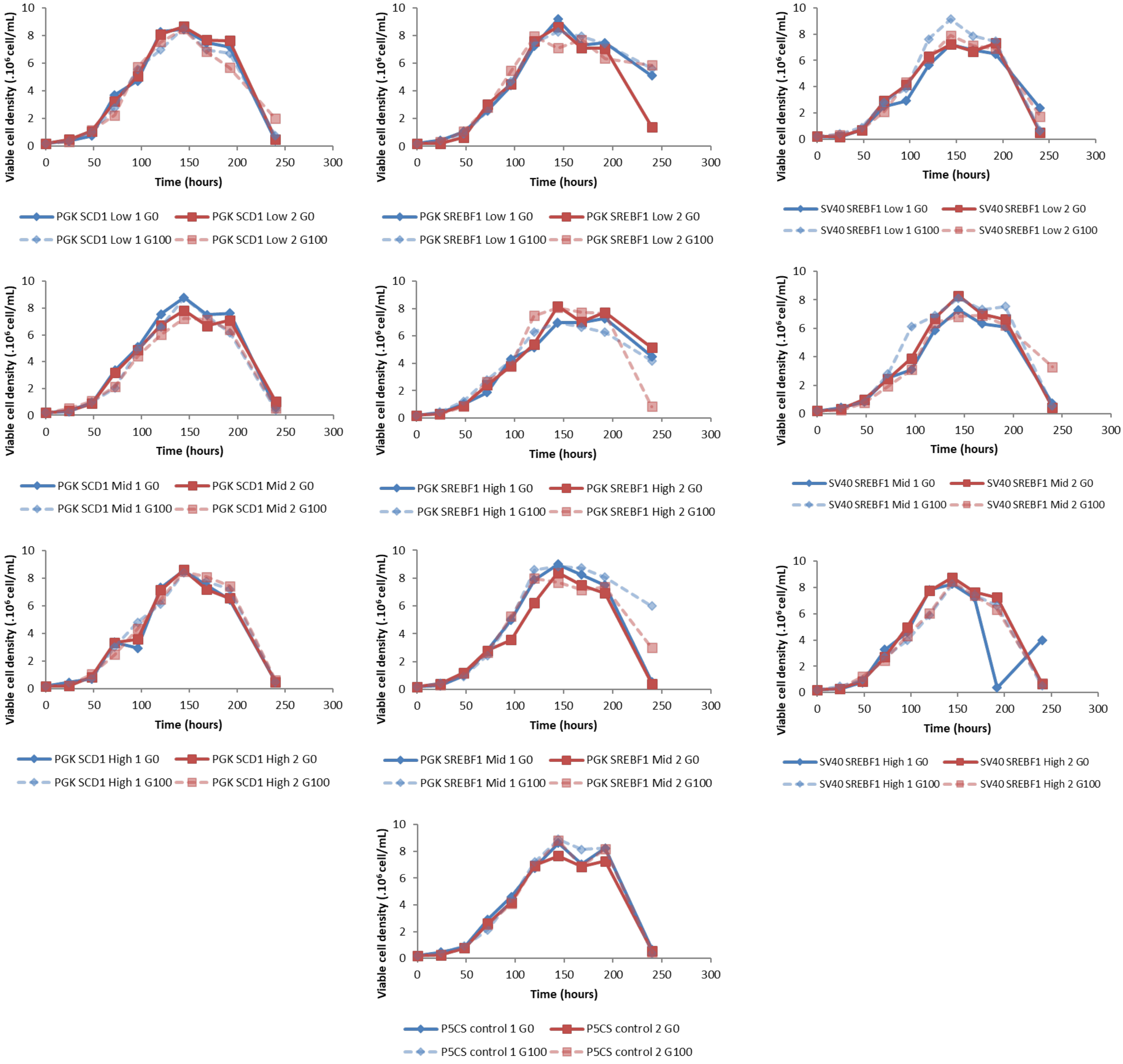

Supplementary Figure 2: Engineered cells were maintained for 100 generations with cells passaged every 3-4 days in the absence of exogenous L-proline and batch culture experiments were run to compare cells derived from the 1st (G0) and 100th generation (G100). The growth profiles were determined by measuring the viable cell concentration of cell pools across batch culture. Control cell pools were generated using constructs containing the P5CS selection marker with no additional gene (J). Cell pools overexpressing either SCD1 or SREBF1 were constructed with a vector containing both the P5CS selection marker and *SCD1* or *SREBF1* where overexpression was driven by either a PGK or SV40 promoter and pools considered either relatively high, low overexpression levels (A-I). Two independent pools were monitored for each condition monitored and growth profiles are distinguished using legends present for each individual graph.
